# Supplementary material for: Serial Llama Immunization with Various SARS-CoV-2 RBD Variants Induces Broad Spectrum Virus-Neutralizing Nanobodies
Source: Vaccines (Basel). 2024 Jan 26;12(2):129. doi: 10.3390/vaccines12020129 (PMC10891761; doi:10.3390/vaccines12020129)
Supplement: Supplementary file 1 [file vaccines-12-00129-s001.zip › vaccines-2775160-supplementary.pdf]

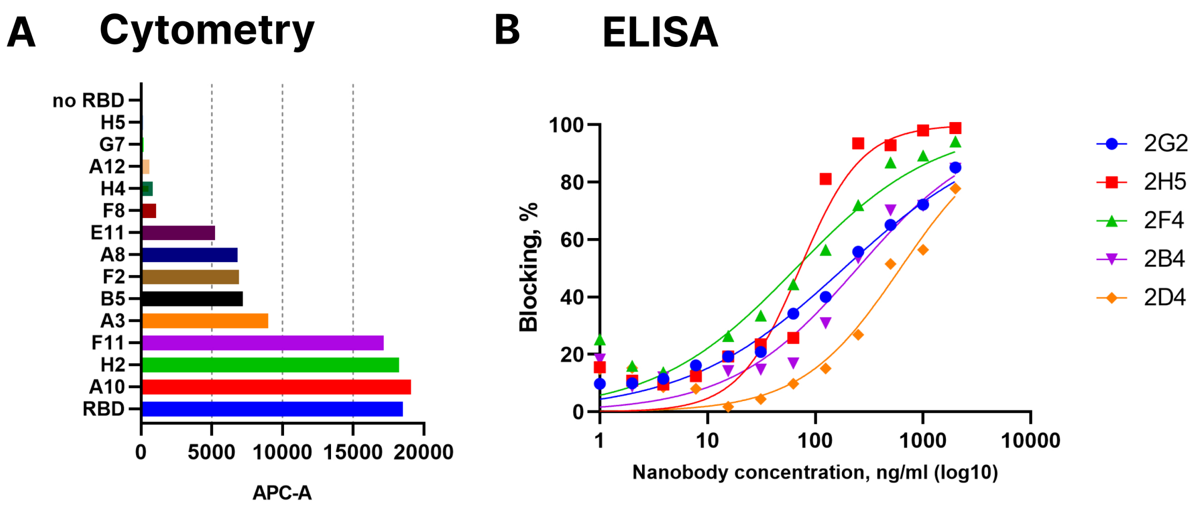

Figure S1. (**A**) Analysis of RBD/ACE2-blocking activity of the nanobodies identified in this study using HEK293T-hACE2 cells. (**B**) Competition ELISA for nanobodies 2F4, 2D4, 2B4, 2H5, and 2G2.
